# Exploring the prospects of artificial intelligence in transforming dental care for special needs groups: mapping the current evidence

**DOI:** 10.1038/s41405-026-00436-x

**Published:** 2026-05-09

**Authors:** Mithun Pai, Shweta Yellapurkar, Kavery Chengappa S, Kalyana C. Pentapati

**Affiliations:** 1 Department of Public Health Dentistry, Manipal College of Dental Sciences Mangalore, Manipal Academy of Higher Education, Manipal, India; 2 Department of Oral Pathology and Microbiology, Manipal College of Dental Sciences Mangalore, Manipal Academy of Higher Education, Manipal, India; 3 Department of Public health Dentistry, Manipal College of Dental Sciences, Manipal Academy of Higher Education, Manipal, India

**Keywords:** Special care dentistry, Dental epidemiology

## Abstract

**Background:**

The integration of Artificial Intelligence (AI) in providing quality dental care to individuals with special needs has been scarcely explored and holds the potential to be transformative. This study aimed to map the current evidence and research gaps on the application of AI tools in special care dentistry.

**Methodology:**

This study was conducted in accordance with the Preferred Reporting Items for Systematic reviews and Meta-Analyses extension for Scoping Reviews (PRISMA-ScR) guidelines to ensure transparency. A systematic search was carried out across PubMed, Google Scholar, Scopus and Web of Science, including studies on AI for dental care of special needs groups published from 2015 to 2025. The data were charted on an evidence map, and study characteristics were evaluated to identify existing evidence and gaps.

**Results:**

Five studies were included in the review. Some of the major themes explored were AI tools used for diagnosis, treatment planning, behavior management, remote consultation and communication assistance for these groups, with one of the five studies assessing dentists' perceptions of their use. This study highlighted the lack of robust evidence and the narrow focus of the existing studies.

**Conclusions:**

This study highlighted the research gap in AI tools for special needs groups and noted the dearth of scientifically and conceptually rigorous studies on the topic. Thus, serving as the preliminary evidence for directing subsequent research on clinical translation of AI tools for special needs groups

## Introduction

Modern-day dentistry has been constantly evolving to extend quality oral care to individuals irrespective of their physical, mental, emotional and environmental limitations. The scope and access to dental care are expanding, with special priority given to those requiring specialized care due to disability and restricted functionality. Special care dentistry caters to these vulnerable populations and is defined by the International Association for Disability and Oral Health as “dentistry for individuals with a disability or activity restriction that directly or indirectly affects their oral health, within the personal and environmental context of the individual” [1]. Persons requiring special needs include physically and mentally challenged persons, immunocompromised persons, those with illnesses, prison inmates, homeless individuals and immigrants. Each of these diverse groups faces personal constraints, such as physical, psychological and cognitive impairments, that impede communication, the delivery of dental treatment and its acceptance. Institutional and social barriers include a lack of policies, structural arrangements and awareness regarding the dental needs of these groups. The overall scarcity of dental care adapted to the requirements of special needs groups, financial and structural constraints in integrating dental and medical care for such individuals, are major hurdles in the delivery of quality dental care [2].

Although conventional dental care systems have superficially addressed these challenges, systems that comprehensively tackle them along the dental care pathway in the interest of these groups are crucial. One such tool that aids in the efficient delivery of dental care to these groups is Artificial Intelligence (AI), which delivers transformative and accurate outcomes that can be included in this realm.

The use of AI in dental caries diagnosis [3], identifying the severity of periodontal disease [4], orthodontic treatment planning [5], diagnosis of oral lesions [6] and automated designing of prosthodontic appliances [7] have been well established. Beyond clinical procedures, AI has also been of considerable use in patient management, epidemiological analysis of dental diseases, disease risk prediction and prevention, dental education and feedback systems [8, 9].

Although AI tools have been clinically used for the general population, their utilization for those needing special care is worth exploring. AI tools can serve as convenient solutions in resolving and simplifying barriers to precise diagnosis, behavior management, tele dentistry for remote access and speech assistance in these groups of patients. The potential of AI to be tailored to specific requirements while expanding dental services to underserved groups makes it a pivotal addition in special care dentistry. However, evidence on the prospects of AI in special care dentistry is fragmented and needs delineation of the knowledge gaps, evaluation of the type of evidence to build a subsequent base for further research. Hence, this study was conceptualized with the objective of mapping the current evidence and the research gap on artificial intelligence tools available for special needs groups and their application in special care dentistry.

## Methods

### Search strategy and selection criteria

This study was conducted in compliance with the Preferred Reporting Items for Systematic reviews and Meta-Analyses extension for Scoping Reviews (PRISMA-ScR) guidelines in order to retain transparent reporting of the methodology [10]. There was no protocol registration done prospectively, nor was risk of bias assessment and quality appraisal of studies conducted for this study consistent with the methodology of evidence mapping. Further, this study only aimed to descriptively map the existing evidence on the topic, without evaluating the methodological quality and rigor of the evidence.

The search strategy involved the use of the keywords along with Boolean operators: (“artificial intelligence”) AND (“Intellectual disability” OR “Physical disability” OR “Medically compromised” OR “Geriatric” OR “Elderly”) AND (“dentistry”). Databases searched included PubMed, Google Scholar, Scopus and Web of Science. Studies from 2015 onwards were included. The time period was limited to the last ten years, considering that the development of artificial intelligence and research in this area has evolved profoundly during the last 10 years.

This study included original studies, reviews and qualitative research. Full-text articles available in English language only were included. Further, a manual search was also conducted for related studies. Those studies that were published in other languages, irrelevant to the scope of this evidence mapping and studies, where the full text was unavailable were excluded. Zotero reference manager was used for importing the retrieved studies, removal of duplicates and management of citations. The articles obtained following the search strategy were screened by reviewer 1 and reviewer 2 independently, and disagreements were resolved through discussions by reviewer 3. The title and abstract screening were followed by review of the full- text and charting of data from the included studies using a pre-conceived data extraction chart manually. The data extracted included study characteristics such as author and year of publication, and study design. Other data included population considered, artificial intelligence tool analyzed, area of interest and conclusion of the study.

The evidence map was created by charting the data obtained from the studies onto a structured extraction format. The variables assessed were the population of special care group considered, the AI tool evaluated and the dental disease or condition for which the AI tool was used. The variable categories were confirmed based on existing literature in medical practice and following familiarization with the domains in the studies included. The special needs groups were categorized based on similarities for comparative assessment, and the AI tools were classified according to their specific functions. The studies under consideration were allotted to one or several categories by reviewer 1 and reviewer 2; any inconsistencies between the two were resolved by reviewer 3. The evidence map was generated as a heat map, with colors corresponding to the number of studies.

#### Ethics approval and consent to participate

 Not applicable.

## Results

**Fig. 1:**
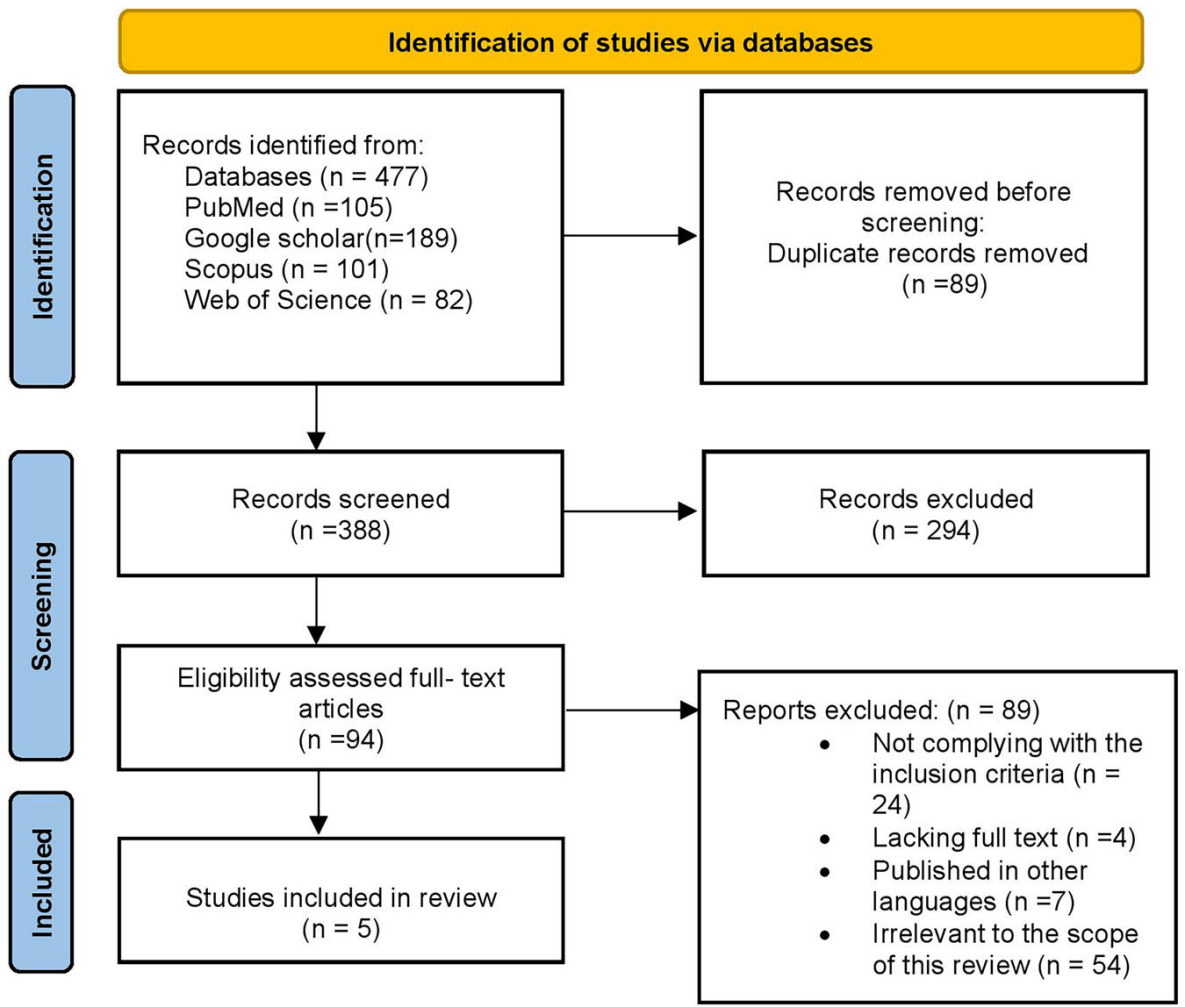
PRISMA flowchart.

The keyword search across databases resulted in 447 studies. Of these, 59 duplicates were removed, resulting in 388 studies. Following the title and abstract screening, 294 studies were eliminated, with 94 studies remaining. Those studies not complying with the inclusion criteria, lacking full text, published in languages other than English and irrelevant to the scope of this study were removed further, resulting in a total of 5 studies that were included in the final evidence mapping (Fig. 1). The domain of AI application in special care dentistry is an emerging and evolving field of research that has largely been unexplored. Hence, the search resulted in a lack of sufficient relevant studies in this specific area of focus. Although only five studies were included, these were the only studies that adhered to the clear inclusion criteria and addressed the major research gap on this topic. Nonetheless, in the discussion, the use of AI in healthcare for special needs groups has been extrapolated to provide context for its dental application. The five included studies were arranged under the subthemes of type of evidence, area of interest, special care group studied, and AI tool analyzed (Table 1).

**Table 1:** Characteristics of the included studies that describe the role of AI in different groups needing special care.

Study no.	Author and year	Study design	Population considered	Artificial intelligence tool analyzed	Area of interest	Conclusion
1	Tiwari et al. [27]	Systematic review	Underserved communities (including elderly persons, head and neck cancer patients, and COVID-19 patients)	AI models such as Machine Learning (ML), Deep Learning (DL), R(Reinforcement) and RL (Reinforcement learning)	Oral, head and neck surveillance	AI tools in smart devices are effective in remote oral health surveillance of underserved communities
2	Al Kheraif et al. [17]	Cross-sectional study	Individuals with mental disabilities (Including cerebral palsy and Down syndrome)	AI-powered tool linked to Galvanic Skin Response (GSR) that measured emotional arousal during dental treatment, coupled with the use of Virtual Reality (VR)	Anxiety, behavior and level of cooperation in the study population	Effective in the behavior management of those with mental disabilities in the dental setup
3	Drafta et al. [12]	Mini review	Vulnerable communities (Rural population, elderly, persons with disabilities, individuals with mental disorders, institutionalized persons, low- resource underserved population)	• AI tool for intraoral image analysis • AI-based tool for analysis of smartphone images • Oral screening using an AI-based tool (Logy AI)	• Diagnostic potential • Patient risk prediction • Automated workflow planning	• 88-95% diagnostic accuracy • High patient satisfaction over 85%
4	Chau et al. [14]	Pilot study	Elderly population in day-care centers	GumAI, a mobile health AI tool for gingivitis screening	• Diagnostic accuracy of gingivitis • Acceptance of the tool among users	High sensitivity and moderate specificity of the tool in comparison with the diagnostic performance of Periodontists.
5	Barnawi et al. [23]	Cross-sectional study	Perception of dentists in treating persons with disabilities	AI tool application in various aspects of clinical dentistry	• Clinical examination • Management of oral complications • Invasive dental procedures • Case history taking • Non- invasive procedure • Behavioral training • Health education • Medical diagnosis • Diagnostic tests • Treatment planning	74.4% of the dentists favored recommending AI use in the clinical dental practice of persons with disabilities

**Fig. 2:**
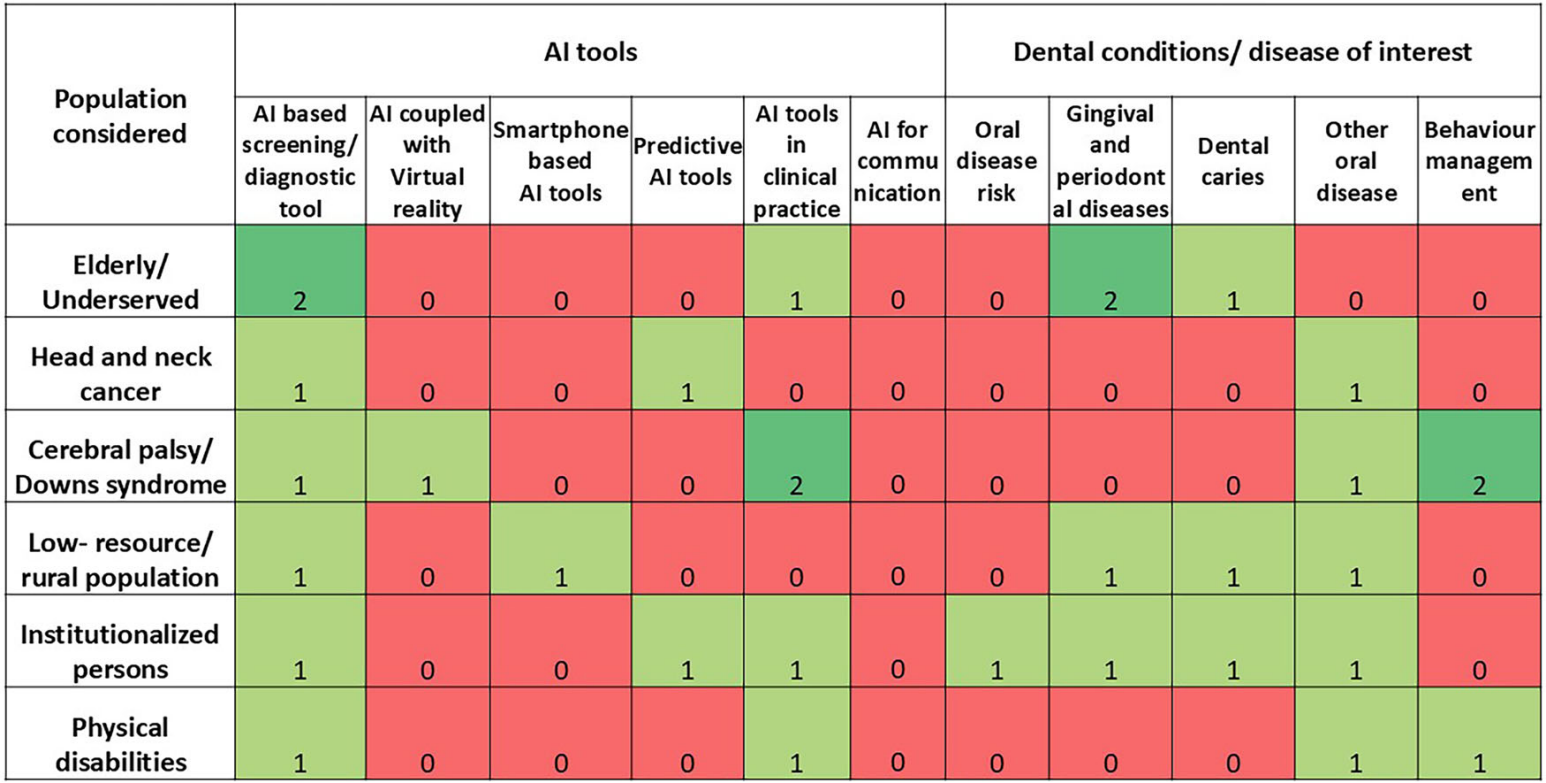
Evidence map showing the distribution of current evidence on AI tools used for special needs groups across specific dental conditions. Color coding: Red—zero studies, Light green—1 study, Dark green—2 or more studies; Numbers in the cells represent the number of studies.

The evidence map showed low, emerging evidence across domains (Fig. 2).

### Type of evidence

Of the studies included, one was a systematic review, two were cross-sectional studies, one was a pilot study, and one was a mini review, with the absence of longitudinal studies and randomized clinical trials on the topic.

### Area of interest

One of the studies assessed oral, head and neck cancer surveillance, another study evaluated behavior, anxiety and cooperation in dental settings. Two other studies examined the diagnostic potential and accuracy, respectively. While one study determined the perception of dentists. The evidence map showed a lack of studies on the use of AI applications in evaluating the risk of oral disease and dental caries in individuals with neurodevelopmental disorders (Fig. 2). Also notable was the absence of studies on the use of AI in the behavior management of head and neck cancer patients, elderly and institutionalized individuals.

### Special care group

The included studies examined the application of AI in varied groups within populations needing special care, such as persons with disabilities, intellectual disabilities, the elderly population and institutionalized persons. The evidence map depicted major studies clustered in the special groups of neurodevelopmental disorders, such as cerebral palsy, Down syndrome and the elderly, underserved groups, assessing different dental conditions in this group (Fig. 2).

### AI tool analyzed

The AI tools and systems that were evaluated include Machine Learning (ML), Deep Learning (DL), Reinforcement Learning (RL) and Reinforcement (R), AI tools used for image analysis, screening of gingival diseases and for measuring emotional changes. All five studies found that the AI tools and systems were effective in their tasks for use among special care groups, with a patient satisfaction of above 85% found in one study. Dentists who used AI for clinical dental practice in case of special needs groups were highly favorable (74.4%) for recommending its use, and 59.2% used AI tools in their special care dental practice. The evidence map showed that most AI applications evaluated were used in screening and diagnostic purposes across the special needs groups (Fig. 2). Most studies also examined AI applications in clinical practice for these groups. One study assessed the use of AI coupled with virtual reality and smartphone-based AI applications.

## Discussion

This study found that the application of AI tools in behavior management, diagnosis, risk prediction, surveillance of oral health and clinical examination was discussed effectively across the included studies. Although few studies were available that studied the direct application of AI, the studies assessed various special groups. The majority of the applications of AI in special care groups were in the fields of screening, diagnosis and behavior management. This study maps existing evidence and identifies research gaps to guide and direct future research in these areas. The themes specify the various areas of dental care where AI tools are utilized in special care dentistry. The following discussion explores these themes, their underlying aspects, potentials and constraints as identified by the existing evidence.

### AI tools for diagnosis and screening

Individuals requiring special care often present with overtly sensitive responses, fearfulness, anxiety and limited cooperation to dental procedures, including examination, that hinder the diagnostic accuracy of dentists [11]. The use of imaging, radiographic analysis and clinical examination for diagnosis, being time and cost-intensive, can further be an impediment in the case of this population for accurate diagnosis. Considering these factors, studies have largely focused on AI tools in the diagnosis and screening of dental diseases [12]. Diagnostic AI tools in these studies have been used to detect incipient dental caries. DentalMonitoring, overjet and Diagnocat are some AI tools that have been assessed to aid in diagnosis at par with clinicians’ diagnosis [12]. Convolutional Neural Networks (CNN) exhibiting greater speed, sensitivity and specificity of diagnosing dental caries, oral precancerous lesions, tooth fracture, periodontal disease, though have been assessed in general populations, their application in special needs groups is largely unexplored [13]. However, one study has assessed GumAI, a smartphone-based tool for screening of gingivitis [14]. Four of the five studies discussed the application of AI in screening and diagnosis of different special needs groups.

The large and diverse training datasets used for AI aid in better accuracy of the diagnosis, provide objective analysis without any bias [3] and have great potential in accurate and quick diagnosis of dental diseases among special needs groups. However, considering these factors, future studies on AI-based screening, diagnostic tools for examining various dental diseases in different special needs groups, and studies on validation of these tools, clinical trials are essential to improve their reliability for real-world usage.

### AI tools for behavior management

Studies have found that special care groups, especially persons with intellectual disabilities, often exhibit anxious and uncooperative behavior during dental procedures [15]. Sedation and general anesthesia have been the mainstay of management in these cases. The use of VR in pain and anxiety management has been shown to correspond with the theory of gate control [16]. When coupled with AI, it can modulate the virtual settings in accordance with the patient’s emotional state, permitting stable behavior control during dental procedures. Of the studies included in the evidence map, two studies discussed behavior management using AI tools in individuals with neurodevelopmental disorders and other physical disabilities. One of the studies examined the use of AI in combination with VR technology among those with cerebral palsy and Down syndrome, to alter their behavior during dental procedures [17]. Despite this being an interventional study, the small sample size and restriction of the study sample to only one special needs group indicate a major lacuna in research on this aspect. Further, the use of other AI tools, such as predictive tools in behavior assessment, has not been sufficiently explored, nor has behavior management in other special needs groups.

### AI tools for communication assistance

Speech and language impairments in those with intellectual disabilities, autistic individuals and elderly populations are a major constraint in not only accessing but also in effectively communicating during dental visits. Various AI-powered conversational agents have been shown to enable persons with cognitive difficulties in effective communication, health management and carrying out daily activities [18]. Through AI-enabled personalized delivery of health educational content, elaborate oral hygiene information can be transformed by natural language processing (NLP) into easy-to-understand instructions for better compliance [19]. Although studies have determined that AI- based chatbots showed promising results in promoting good health practices among the general population [20], their adaptation for individuals with special needs requires various considerations. The evidence map showed a complete lack of studies on the direct application of AI in addressing communication constraints for simplifying dental treatment procedures among special needs groups. The lack of studies on this domain specifically for those with intellectual, speech, vision and hearing disabilities is a critical area warranting further research in real- world settings.

### AI tool for treatment planning

In case of individuals with special care needs, their medical history, response to dental treatment, level of understanding, oral hygiene practices and clinical conditions need to be taken into consideration to devise a tailored dental treatment plan [21]. The process being resource-intensive and time-consuming, a dentist’s experience and clinical acumen are critical to making the decision. Research in this area has shown that AI algorithms enable rapid planning, along with features such as predictive analytics, which help to prioritize specific treatments based on dental disease risk [22]. Studies have shown that AI applications such as Previsoft and Overjet allow for risk characterization of patients based on dental conditions, aiding in treatment planning [12]. However, the evidence map showed three studies utilized AI in clinical practice, with only one study discussing AI for treatment planning of the special needs groups [23]. The scope of incorporating AI tools to deliver personalized, swift and simplified dental treatment plans, catering to the special needs, needs to be explored with longitudinal studies.

### AI for remote consultation

The availability of dental specialists providing dental care to a special care group of patients is scarce, and hence, access to dental care may be limited. Additionally, financial constraints, logistic barriers and caregivers’ limitations can hinder access to dental care [23]. Tools such as Apple Tree Dental, which provide mobile-supported tele-dentistry, have simplified timely dental care for persons with disabilities by addressing these accessibility concerns [12]. While a systematic review synthesized many such potential AI-based applications, it also highlighted that these tools are in the pilot or early experimental stages, lacking standard frameworks for their integration into dental care [24].

### Dentists’ perception of adopting AI in special care dentistry

The perception, attitude and awareness of dentists regarding AI in providing dental care to the special needs groups is critical to understand the shortcomings and potential that need to be harnessed further. Hence, this study also mapped evidence from a study that assessed the perception of dentists. Barnawi et al., in their study conducted among dentists of Saudi Arabia, found that 59.2% of the dentists incorporated AI in clinical dental practice [23]. The study further found that the majority of applications of AI were in treatment planning (43.7%). Inference from the evidence map indicated the study evaluated AI-based screening and clinical tools among those with intellectual and physical disabilities. This study also assessed the application of AI in the behavior management of those with special needs. More than half of the study participants had a positive perception of AI in special care dentistry. Previous training in AI was one of the strongest predictors of its use. The study implied that developing a standard AI framework and training dental practitioners in its use could help improve its adoption in special care dentistry [23].

### AI in assistive technologies with potential for application in special care dentistry

The scope of AI in assistive healthcare technology has been studied, and it has shown various applications. Their use has been majorly included in assistive functions such as aids for navigating, language assistance and cognitive support [25]. Integrating AI, the Internet of Things can further enhance its usability for persons needing special care. Wearable smart devices that monitor health parameters can aid in medical and behavioral management during dental visits. Another assistive technology, the Environmental Control System (ECS), facilitates physically or intellectually disabled persons to manage and interact with their environment. Integrating AI and ECS to examine behavioral and emotional changes that enable altering the dental clinic environment accordingly could ease the anxiety of such patients during dental visits [26].

### Potential clinical implications

Based on the studies included in this study, the evidence base to support the clinical application of AI tools is lacking in major aspects. However, the existing evidence suggests that AI tools are presently considered as supplemental tools utilized at various levels of dental care as opposed to being independent decision-making, functioning systems. Evidence mapping indicates that initial stage studies have explored the potential use of AI tools for behavior management, risk prediction and diagnosing dental disease in special needs groups. However, there is a complete lack of scientifically robust interventional studies, longitudinal studies and questionnaire-based studies assessing the real-world application of AI tools for individuals with special needs. Moreover, potential areas of clinical application for dental care of special needs groups, such as AI tools for communication, AI tools for predicting oral disease risk and predicting behavioral changes, have not been adequately studied. Hence, this evidence map highlights the research gaps and the prospective areas of clinical application for investigating further.

### Ethical challenges

Considering the application of most AI tools involves input of private data and data on personal health, ethical and safety concerns are certain. Integration of AI necessitates procedures such as data anonymization of personal data of users and algorithmic transparency to ascertain that the functioning of AI systems is ethical and safe. Compliance of such systems with regional and international regulations, such as the Digital Personal Data Protection Act, General Data Protection Regulation (GDPR), is essential for the ethical and safe performance of AI tools. Further studies could incorporate an analysis of the ethical aspects of using AI tools in these groups.

### Limitations and recommendations

The findings of this study highlight the varied potential of AI in transforming oral care delivery for those with special needs. However, the limitations of this study need careful acknowledgment in analyzing these results. The dearth of scientifically robust studies in this context not only raises the need for more studies in this area but also studies with larger populations, varied special needs groups and rigorous study design, examining long- term outcomes. AI tools evaluated in the included studies were diverse, hampering comparison and lacked external validation among larger special needs groups. The real-world application of these tools and applications has been scarcely studied, raising concerns about their clinical and practical utility. One of the major concerns is ethical challenges and threats to patients’ privacy; ambiguity in this aspect warrants research on ethically and legally compliant clinical implementation of these AI tools. Studies also need to explore the impact of AI tools in special care dentistry on the oral health-related quality of life of these individuals. With evidence on AI in dentistry evolving, there is a need to broaden the scope of research for the application of AI in special care dentistry.

### Recommendations for future research

The dearth of scientifically robust studies on AI in dental care of special needs groups warrants more studies in this direction. More primary studies that longitudinally assess the implementation of such tools and in real-world scenarios are crucial for their adoption in special needs groups. Moreover, the inclusion of larger and more varied groups of individuals needing special care can validate the generalization of these AI tools. Further research on the ethical and legal validity of these AI tools for special needs individuals, which retain the privacy and sovereignty of individuals seeking care, is crucial.

## Conclusion

This evidence mapping study highlights the large research gap, with currently only early-stage and exploratory studies on the topic. Longitudinal and scientifically validated research on the application of AI tools in dental care for those with special needs is of pressing priority. Clinical trials and real-world studies, along with patient-centered outcomes, are primary for clinical implementation of these tools. It is imperative to address the ethical and implementation challenges of AI tools at the policy level to ensure their practical use. Moreover, integrating AI into dental education should form the premise of incorporating AI in the dental care of the special needs population. Overall, evidence from this study provides a perspective of the gaps, prospects and high-impact areas that can serve as the foundation for subsequent investigations on AI tools for special needs groups.

## Data Availability

No datasets were generated or analyzed during the current study.
